# Early do-not-resuscitate orders in intracerebral haemorrhage; frequency and predictive value for death and functional outcome. A retrospective cohort study

**DOI:** 10.1186/1757-7241-20-36

**Published:** 2012-05-25

**Authors:** Marco Brizzi, Kasim Abul-Kasim, Mattis Jalakas, Eufrozina Selariu, Hélène Pessah-Rasmussen, Elisabet Zia

**Affiliations:** 1 Department of Neurology, Malmö, Sweden; 2 Department of Radiology, Skåne University Hospital, Ing 52, 205 02, Malmö, Sweden

**Keywords:** Intracerebral haemorrhage, Prognosis, Functional outcome, Mortality, Do not resuscitate orders

## Abstract

**Introduction:**

In former studies from North America early Do-Not–Resuscitate orders (DNR orders) in patients with intracerebral haemorrhage (ICH) had negative prognostic impact on mortality. The influence of DNR orders on functional outcome and whether DNR orders are grounded on relevant patient characteristics is unknown. We aimed to determine the frequency and predictive factors of DNR-orders and its association to prognosis, in ICH patients, in Scandinavia.

**Methods:**

In 197 consecutive ICH patients admitted to Skåne University Hospital, Malmö, Sweden, between January 2007 and June 2009, information of the presence of DNR orders within 48 hours, clinical and radiological characteristics was retrieved by review of patient medical journal and computed tomography scans. Determinants of DNR-orders, one-month case fatality and bad functional outcome (modified Rankin Scale, grade 4–6) were assessed by logistic regression analysis.

**Results:**

DNR orders were made in 41% of the cases. After adjustment for confounding factors, age ≥ 75 years (Odds Ratio (95% confidence interval) 4.2(1.8-9.6)), former stroke (5.1(1.9-3.1)), Reaction Level Scale grade 2–3 and 4 (7.0(2.8-17.5) and (4.1(1.2-13.5), respectively) and intraventricular haemorrhage (3.8(1.6-9.4)) were independent determinants of early DNR orders. Independent predictors of one-month case fatality was age ≥ 75 years (3.7(1.4-9.6)) volume ≥ 30 ml (3.5(1.3-9.6)) and DNR orders (3.5(1.5-8.6)). Seizure (6.0(1.04-34.2) and brain stem hemorrhage (8.0(1.1-58.4)) were related to bad functional outcome, whereas early DNR order was not (3.5(0.99-12.7)).

**Conclusions:**

Well known prognostic factors are determinants for DNR orders, however DNR orders are independently related to one-month case fatality. In addition to improvements of the local routines, we welcome a change of attitude with an enhanced awareness of the definition of, and a more careful approach with respect to DNR orders.

## Introduction

Intracerebral haemorrhage (ICH) accounts for ≈ 10% of acute stroke cases in western communities [1,2]. One-month case fatality rate is high, 25-50% [3-5], as compared to 10% for cerebral infarction [6]. With this knowledge of poor prognosis, physicians who receive acute ICH patients may raise questions about level of care decisions, sometimes with a palliative approach already in an early stage, in order to avoid exposing the patient from unnecessary suffering. Stroke-unit care, including precautions against aspiration, fever, high blood pressure and other factors that may contribute to early neurological deterioration, is well established for ICH patients in the acute phase [7]. Initial aggressive care, including mechanical ventilation, may reduce mortality and disability for some patients, although many will have severe disability despite these efforts [8,9].

Haemorrhagic characteristics and age are important prognostic factors [3,5]. However it has been shown, in North American studies, that Do- Not –Resuscitate orders (DNR orders) within 24 hours from admission predict mortality after ICH [10-12]. The impact of DNR order on functional outcome is not known. There are large differences between hospitals practice of DNR orders [11,13] and differences in practice between countries as well are plausible. We aimed to determine the frequency and determinant factors of DNR orders within 48 hours from admission to hospital, and its association with one-month case fatality rate and three month functional outcome in patients with ICH at a University Hospital in Scandinavia.

## Methods and materials

### ICH case ascertainment

This study includes all consecutive cases with spontaneous intracerebral haemorrhage admitted to the University Hospital of Skåne, Malmö, between January 2007 and June 2009. The hospital is the only one serving the population of Malmö (n ≈ 300 000), and the volume of stroke patient admissions is one of the largest in Sweden (n ≈ 800/year). Cases with International Classification of Diseases (ICD) code I61.1-9 (intracerebral haemorrhage), I63.0-9 (cerebral infarction) and I64.0-9 (unspecified stroke) were identified from the National Hospital Discharge register [14]. A research nurse and a neurologist validated the diagnosis by review of medical records. Stroke was defined following the WHO´s definition [15]; ICH was considered when CT or autopsy showed intraparenchymal blood in the brain, irrespectively of potential underlying vascular malformation. Traumatic haemorrhages were not included. Out of an eligible cohort of 203 subjects, 7 subjects were excluded (acute care at other hospital (n = 5) and tumor (n = 1)) and 197 were included in this study.

### DNR order

Information on DNR orders, and time of DNR orders in relation to admission, were retrieved by review of the patients’ journal. Doctor, nurse and paramedics documentations from the emergency department, intensive care unit (ICU), neurological department and/or other relevant ward were reviewed.

The hospital definition of DNR order is no resuscitation in case of cardiac arrest, without other limitations of care. The local routine during the study period was to document the decision in the patients´ journal (usually in the medication list, the doctors documentation and/or in the observation charts) assessed as DNR order present or not present. In this study, we defined early DNR order as documented information of DNR order within 48 hours from admission to the hospital and lack of documentation was assessed as no DNR order.

### Baseline characteristics

Information of seizure in the acute phase (≤ 48 h), care at intensive care unit (ICU) or referral to neurosurgery department, comorbidity (malignancy, heart and/or lung disease or other severe disease with impact on dependency and daily activity), DNR order prior to ICH event, and time from event to arrival to hospital, was retrieved from patient journal.

Information of diabetes (diet or pharmacological treatment), treatment with antithrombotics (Aspirin, Clopidogrel, Dipyridamole), warfarin treatment, hypertension medication and smoking habit (current smoker/non smoker), on admission, was retrieved from patient medical journal. Level of consciousness on admission was assessed according to the Reaction Level Scale (RLS 85 scale) [16]. The RLS scores were categorized into three groups: RLS score = 1 (alert), score 2 to 3 (drowsy) and score ≥ 4 (unconscious). Time from stroke to admission to hospital was assessed as <2, 2–24, and > 24 hours.

### Evaluation of the radiological studies

Computed tomography (CT) was performed on admission of all patients included in the study. The images were evaluated by neuroradiologists with experience in stroke. The haemorrhage location, midline shift and intraventricular haemorrhages were determined. Haemorrhage location was classified into five categories: lobar (predominantly cortical and subcortical white matter), deep (basal ganglia, internal capsule, periventricular white matter), brainstem, cerebellar and multiple. The haemorrhage volume was calculated from the three measured diameters by using the following formula (width x length x height x 0,5) [17].

### Outcome

Information of date of death was assessed from the National Cause of Death register [18]. All cases at the clinic with validated stroke diagnosis are routinely registered in a National Stroke Register [19]. Functional outcome as measured by Modified Rankin scale (mRS) at three months, was achieved by converting answers from 5 items from the three months follow-up questionnaire in this register [20]. Bad outcome was defined as mRS grade 4–6 (mRS 4 = moderately severe disability; unable to walk without assistance and unable to attend to own bodily needs without assistance, mRS 6 = dead).

### Statistics

Differences in baseline characteristics in patients with DNR orders, as compared to those without, were calculated by Chi square and one-way Anova test for categorical and continuous variables, respectively (Table 1).

**Table 1:** Baseline characteristics for ICH patients with- and without early DNR order

	**Total**	**Early DNR order**	**No early DNR order**	***P value*(early DNR vs not)**
**n**	197	80 (40.6)	117 (59.4)	
**Men**	88 (44.7)	31 (38.8)	57 (48.7)	
**Women**	109 (55.3)	49 (61.3)	60 (51.3)	0.190*
**Age, mean ± SD**	73 ± 14	78 ± 11	70 ± 14	<0.001
**Hypertension medication**	123 (62.4)	47 (58.8)	76 (65.5)	0.369
**Current smoker**	21 (10.7)	8 (10.4)	13 (11.3)	1.0
**Atrial fibrillation**	23 (11.7)	11 (13.8)	12 (10.3)	0.503
**Former stroke**	39 (19.8)	25 (31.3)	14 (12.1)	0.002
**Diabetes**	23 (11.7)	7 (8.8)	16 (13.7)	0.369
**Warfarin**	17 (9.0)	7 (9.5)	10 (8.8)	1.0
***Haemorrhage site***				
**Lobar**	81 (41.1)	31 (38.8)	50 (42.7)	
**Deep**	88 (44.7)	37 (46.3)	51 (43.6)	
**Brainstem**	11 (5.6)	4 (5.0)	7 (6.0)	
**Cerebellar**	16 (8.1)	7 (8.8)	9 (7.7)	
**Multiple**	1 (0.5)	1 (1.3)	0 (0)	ns
**Midlineshift**	51 (25.9)	38 (47.5)	13 (11.1)	<0.001
**Intaventricular haemorrhage**	66 (33.5)	45 (56.3)	21 (17.9)	<0.001
***Volume***				
**≤ 30 ml**	146 (74.1)	99 (58.8)	47 (84.5)	
**> 30 ml**	51 (25.9)	18 (41.3)	33 (15.4)	<0.001
**Mean ± SD/** **Median**	26.5 ± 38.6/9.2	41.4 ± 47.1/22	16.4 ± 27.3/7	<0.001
***Time from ICH to admission***				
**< 2 hours**	88 (44.9)	48 (41.0)	40 (50.6)	
**2-24 hours**	78 (39.8)	40 (42.7)	28 (35.4)	
**> 24 hours**	30 (15.3)	19 (16.2)	11 (13.9)	0.414
***RLS on admission***				
**1 (alert)**	99 (50.3)	19 (23.8)	80 (68.4)	
**2-3 (drowsy)**	63 (32.0)	39 (48.8)	24 (20.5)	
**≥4 (unconscious)**	35 (17.8)	22 (27.5)	13 (11.1)	<0.001
**Pre-ICH comorbidity**	55 (27.9)	30 (37.5)	25(21.4)	0.01
**Seizure**	19 (9.6)	11 (13.8)	8 (6.8)	0.140
**ICU care**	18 (9.1)	9 (11.3)	9 (7.7)	0.454
**Neurosurgery care**	12 (6.1)	0 (0)	12 (10.3)	0.002
**Prior DNR decision**	3 (1.5)	2 (2.5)	1 (0.9)	0.568

· women as compared to men.

To explore predictive variables for DNR orders and outcome (one month case fatality rate and mRS at three months), logistic regression model was used. DNR order, fatal outcome and bad functional outcome, (mRS grade 4–6) respectively, were assessed as dependent variable. In the first step, each baseline variable (Table 1) was entered individually in the models. Factors with p < 0.2 in this first step were considered as potential confounding factor, and were included in the multivariate models (Table 2, 3 and 4). Due to the well-known high early mortality in ICH patients, only patients who survived one month were included in the analysis of outcome at three months in order to evaluate functional outcome, rather than mortality.

**Table 2:** Determinants of early DNR order in ICH patients (multivariate analysis)

	**OR (95% CI)**
**Age**	
**< 75 yrs**	1
**≥ 75 yrs**	4.2 (1.8-9.6)
**RLS grade**	
**1 (alert)**	1
**2**–**3 (drowsy)**	7.0 (2.8-17.5)
**≥4 (unconscious)**	4.0 (1.2-13.5)
**Comorbidity**	1.3 (0.6-3.1)
**Seizure**	1.1 (0.3-4.0)
**Former stroke**	5.1 (1.9-13.4)
**Volume**	
**< 30 ml**	1
**≥30 ml**	1.7 (0.6-4.7)
**Midlineshift**	2.7 (0.9-7.7)
**Intraventricular haemorrhage**	3.8 (1.6-9.4)
**Gender**	
**Men**	1
**Women**	1.9 (0.9-4.2)

**Table 3:** Determinants of 1 month Case Fatality Rate in ICH patients (multivariate analysis)

	**OR (95% CI)**
**Age**	
**< 75 yrs**	1
**≥ 75 yrs**	3.7 (1.4-9.6)
**RLS**	
**1 (alert)**	1
**2**–**3 (drowsy)**	1.9 (0.7-5.1)
**≥4 (unconscious)**	3.0 (0.9-10.4)
**Comorbidity**	1.6 (0.7-3.9)
**Diabetes**	0.4 (0.1-2.0)
**Hypertension medication**	0.5 (0.2-1.1)
**Volume**	
**< 30 ml**	1
**≥30 ml**	3.5 (1.3-9.6)
**Midlineshift**	1.8 (0.6-5.6)
**ICU care**	4.1 (0.9-18.5)
**Early DNR order**	3.5 (1.5-8.6)

**Table 4:** Determinants of mRS-score ≥ 4 at 3 months in ICH patients (one-month survivors, multivariate analysis)

	**OR (95% CI)**
**RLS**	
**1 (alert)** **2**–**3 (drowsy)**	1
**≥4 (unconscious)**	2.8 (0–9.8.8) 3.2 (0.4-26.6)
**Seizure**	6.0 (1.04-34.2)
**Former stroke**	1.8 (0.6-5.8)
**Hypertension medication**	0.3 (0.1-0.9)
**Midlineshift**	0.5 (0.01-2.7)
**Heamorrhage location**	
**Lobar**	1
**Deep**	2.8 (0.9-8.8)
**Brainstem**	8.0 (1.1-58.4)
**Cerebellar**	1.6 (0.2-13.8)
**Care at neurosurgery dept**	2.7 (0.3-28.9)
**Early DNR order**	3.5 (0.99-12.7)

Information of functional outcome at three months was missing in thirteen cases, and those were excluded from the analysis. All comparisons were two-sided and a 5% level of significance was used. Statistical analysis were conducted by computer software SPSS (version 18.0).

## Results

In total, 197 ICH patients were included in the study. Mean age was 73 ± 14 years, and 45% were men. In 41% of the cases, DNR orders were made within 48 hours from arrival to the hospital, and 36% of the cases had a DNR order within 24 hours from arrival. Baseline characteristics in relation to the presence of DNR order (within 48 hours) or not, are presented in Table 1.

### Determinants of early DNR order

Factors related to DNR order were age ≥ 75 years (Odds Ratio (OR) 95% confidence interval (95% CI) 2.7 (1.5-4.9)), former stroke (OR 3.3 (1.6-6.9)), RLS grade 2–3 and 4 (OR 6.8(3.4-14.0) and 7.1(3.0-16.6), respectively), volume ≥ 30 ml (OR 3.9(2.0-7.6)), midlineshift (OR 7.2 (3.5-14.9) and the presence of intraventicular haemorrhage (OR 5.8 (3.1-11.2)). After adjustment for confounding factors (Table 2), age ≥ 75 years, former stroke, RLS grade 2–3 and 4 and the presence of intraventricular hemorrhage were independent predictors of DNR order.

### DNR order as prognostic determinant

**Figure 1:**
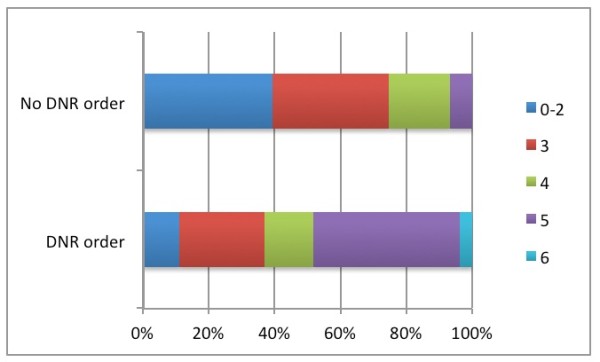
**Three months functional outcome as measured by modified Rankin Scale in ICH patients with respectively without early DNR order.** Only one month survivors are included (n = 118).

One month case fatality rate was 33.4% (66/197 ICH cases). Of those who survived one month, 30.5% had bad functional outcome at three months (40/118, 13 survivors with missing information of mRS). Mean age of one-month survivors was 71 ± 14 years as compared to 77 ± 11 years in nonsurvivors (p = 0.001). Functional outcome, including late mortality, in relation to DNR order for the entire study population is presented in Figure 1. Of those with DNR order, 37% had good functional outcome (mRS grade 0–3) as compared to 75% in those without a DNR order.

Factors related to elevated risk of one month case fatality were age ≥ 75 years (OR 2.5(1.4-4.7)), comorbidity (OR 2.7(1.1-3.9)), RLS grade 2–3 and 4 (OR 4.5 (2.1-9.4 and 10.7(4.4-25.1), respectively), volume ≥30 ml (OR 8.1(4.0-16.5)), midlineshift (OR 6.3(3.1-12.6)), intraventricular haemorrhage (OR 6.2(3.2-12.0)), ICU care (OR 4.6(1.7-13.0)) and DNR order (OR (9.3 (4.7-18.4)). There was a reverse relation ship between hypertension medication and one month CFR (OR 0.4 (0.2-0.8). After adjustment for confounding factors, age ≥75 years, volume ≥ 30 ml and DNR order were independent predictors of one month CFR, whereas having hypertension medication was protective for death at one month (Table 3).

In the univariate analysisis, former stroke (OR 3.3(1.3-8.4)), seizure (OR 6.3 (1.6-8.4)), RLS grade 2–3 and 4 (OR 4.0(1.6-9.8) and 10.0 (2.3-42.1), respectively), midlineshift (OR 3.5 (1.1-10.6)), deep haemorrhage location (OR 2.5(1.04-6.2)) and DNR order (OR 5.0 (2.0-12.5)) were related to bad functional outcome at three months. After adjustment for confounding factors, hypertension medication was inversely related to bad functional outcome whereas seizure in the acute phase and brainstem haemorrhage were independent risk factors for bad functional outcome. DNR order was not an independent risk factor for bad functional outcome (OR 3.5 (0.99-12.7))(Table 4).

## Discussion

In a consecutive, unselected large ICH population in Sweden, early DNR orders were made in 41% of the cases, and independently predicted one-month case fatality. To the best of our knowledge, this has not been previously shown in a non-American study.

Initial triage of ICH patients is a difficult challenge. Perhaps with intention to save the patient from suffering, there might be a tendency to take drastic decisions of level of care during the first few days, and sometimes already in the emergency room at arrival to the hospital. In our study 36% of the patients had a DNR order already within 24 hours from arrival to hospital. Despite differences in care traditions (for example, 9% of our patients had ICU care as compared to 30% in California [11]), the results are in accordance with those from North America were DNR orders within 24 hours were made in 25-35% of the patients [11,12,21].

Underestimation of the chances for reasonable outcome after stroke may contribute to withhold of life sustaining therapies [8]. Early DNR orders may turn out to be a justification for an over all decrease in aggressiveness of care, beyond no attempt at resuscitation in the event of cardiopulmonary arrest. After adjustment for well-known predictive factors, DNR decision was related to a 3.5 fold increment of risk of death. One-month case fatality rate was 61% in those *with* a DNR decision, as compared to 14% in those without. This might of course reflect the fact that the physician had considered the well-known prognostic factors [22] when making a DNR order. In fact, high age, low conscious level, former stroke and intraventricular haemorrhage were all independent determinants of a DNR decision. It is possible that a DNR order is a surrogate marker of recognized prognostic factors. Nevertheless, DNR orders were independently related to one month CFR after adjustment for these factors. Prognostication in the acute phase of ICH is difficult [8] and validated predictive models developed to stratify risk of death following ICH might give a false sense of accuracy when DNR status is not considered. Further on, these models are difficult to rely on in the decision process of DNR orders on individual patients [21,23].

In this study DNR orders did not independently predict bad functional outcome (p value = 0.051), although one could speculate that larger sample size would have strengthened the results. As presented in Figure 1, one month survivors from the DNR group had as expected a worse functional outcome as compared to the group without DNR order, 59% mRS grade 4–5 versus 25% (Figure 1). Nevertheless, 37% of the patients with DNR orders had good functional outcome, confirming that functional outcome after ICH is awkward to predict in individual patients [21,23].

With respect to the difficulties of correct evaluation of all possible factors influencing initial performance of an acute ICH patient, and the uncertainty in early prognostication, the American ICH guidelines from 2010 strongly suggest aggressive initial therapy and to avoid DNR orders or withdrawal of support during the first few days, as necessary precautions for providing fair chances to good outcome for the individual patient [23]. This may appear as a challenge with respect to acute care organization and intensive care life support for the unconscious ICH. However, it should be kept in mind that this patient group accounted for 18% in our study, of which in particular those without control of airway and breathing functions would be considered for ICU care, and in most cases with one or a few days of stabilization. The topic was also highlighted in the National Guidelines for Stroke care, Sweden, in 2009 and there have been changes in the local routines of documentation of DNR order for all diagnosis since 2006. However, despite this increased awareness, the writers’ experience is that an early DNR decision is still not rare, and with the consequences of less aggressive care overall. During the study period, it was not obligate to specify the causes of a DNR order, which often was made by a single physician. The latest local routines require the physician to make a decision of DNR order or not on admission, including a documentation of the causes the decision is based on, and in accordance with whom. A systematic approach to DNR orders, including a new evaluation daily might improve the routines further.

A limitation of this study is its retrospective nature. However, with respect to difficulties in identification of DNR decision (sometimes handwritten scanned notes), we might have underestimated the rate and hence the main results (rate of DNR decision and prognostic impact). We lack information of the specialty and experience of the decision-making physicians, factors that might influence the frequency of DNR orders. Of the one-month survivors, only 1 of 118 patients died within three months, well in accordance with other studies showing that early mortality after ICH occurs within the first days ([5,24]. The rate of death, hemorrhage characteristics and level of consciousness is in line with those from other studies with unselected ICH cases [3,25].

## Conclusion

At the University Hospital in Malmö, with one of the largest volume of acute stroke patients in Sweden, early DNR orders are frequent among ICH patients. Well known prognostic factors are determinants for a DNR decision, however DNR orders are independently related to one-month case fatality and a substantial amount of patients with early DNR orders survived with good functional outcome. In addition to improvements of the local routines, we welcome a change of attitude with an enhanced awareness of the definition of, and a more careful approach with respect to DNR orders.

## Abbreviations

ICH, Intracerebral haemorrhage; DN, Do not resuscitate; RLS, Reaction level scale; mRS, modified rankin scale; ICU, Intensive care unit; CT, Computed tomography; OR, Odds ratio; CI, Confidence interval.

## Competing interests

The authors declare that they have no competing interests.

## Authors’ contributions

EZ and MB conceived the study and took responsibility for the design and implementation of the study. EZ, MB, and MK assessed clinical information by review of patient journals. HPR was responsible for the diagnosis validation process and follow-up process for the national stroke register. KAK and ES assessed neuroradiological information. All authors had full access to all data and took responsability for their integrity. EZ did the statistical analysis and drafted the manuscript. All authors critically revised the manuscript and contributed with intellectual comments and approved the final version for publication. All authors declare that they accept full responsability for the conduct of the study and controlled the decision to publish. All authors read and approved the final manuscript.
